# Supplementary material for: Clinicopathological Characteristics of Ovarian and Breast Cancer in PALB2, RAD51C, and RAD51D Germline Pathogenic Variant Carriers
Source: Genes (Basel). 2025 May 2;16(5):556. doi: 10.3390/genes16050556 (PMC12111020; doi:10.3390/genes16050556)
Supplement: Supplementary file 1 [file genes-16-00556-s001.zip › genes-3594266-supplementary.pdf]

	PALB2<br>*n*=61	*RAD51C*<br>*n*=11	*RAD51D*<br>*n*=4
Breast cancer	50 (81.9%)	3 (27.3%)	1 (25.0%)
Ovarian cancer	0	4 (36.4%)	2 (50.0%)
Skin cancer	4 (6.6%)	2 (18.2%)	1 (25.0%)
Basal cell carcinoma	1 (1.6%)	1 (9.1%)	1 (25.0%)
Squamous cell carcinoma	1 (1.6%)	-	-
Melanoma	2 (3.3%)	1 (9.1%)	-
Endometroid cancer	1 (1.6%)	-	-
Lung cancer	1 (1.6%)	-	-
Stomach cancer	1 (1.6%)	-	-
Colon-rectal cancer	1 (1.6%)	-	-
Other	2 (3.3 %)	-	-
Unknown	1 (1.6%)	3 (27.3%)	-

Table S1. Prevalence of primary cancer types by GPV
